# Crystal structure of the mixed methanol and ethanol solvate of bis­{3,4,5-trimeth­oxy-*N*′-[1-(pyridin-2-yl)ethyl­idene]benzohydrazidato}zinc(II)

**DOI:** 10.1107/S2056989020000857

**Published:** 2020-02-06

**Authors:** Kateryna Znovjyak, Igor O. Fritsky, Tatiana Y. Sliva, Vladimir M. Amirkhanov, Maksym Seredyuk

**Affiliations:** a Department of Chemistry, Taras Shevchenko National University of Kyiv, Volodymyrska Street 64, Kyiv 01601, Ukraine; b UkrOrgSyntez Ltd, Chervonotkatska Street 67, Kyiv 02094, Ukraine

**Keywords:** crystal structure, zinc(II) complex, hydrazone derivatives, Hirshfeld analysis

## Abstract

The unit cell of the title compound, [Zn(C_17_H_18_N_3_O_4_)_2_]·CH_4_O·C_2_H_6_O, contains two complex mol­ecules related by an inversion centre, plus one methanol and one ethanol solvent molecule per complex molecule. In each complex, two deprotonated pyridine aroylhydrazone ligands {3,4,5-trimeth­oxy-*N*′-[1-(pyridin-2-yl)ethyl­idene]benzohydrazide} coordinate to the Zn^II^ ion through the N atoms of the pyridine group and the ketamine, and, additionally, through the O atom of the enolate group. In the crystal, dimers are formed by π–π inter­actions between the planar ligand moieties, which are further connected by C⋯O and C⋯C inter­actions. The inter­molecular inter­actions were investigated using Hirshfeld surface analysis and two-dimensional fingerprint plots, revealing that the most important contributions for the crystal packing are from H⋯H (44.8%), H⋯C/C⋯H (22.2%), H⋯O/O⋯H (18.7%) and C⋯C (3.9%) inter­actions.

## Abstract 2

The title compound belongs to the class of neutral pyridine aroylhydrazone complexes. In the crystal, π–π stacking leads to dimerization of the complex mol­ecules, which, in turn, are connected by weak C⋯O and C⋯C inter­actions, thus giving rise to a supra­molecular layered architecture.

## Chemical context   

Aroylhydrazones are an attractive class of ligands exhibiting coordination versatility toward a wide range of metals, particularly 3*d* transition metal ions (Bernhardt *et al.*, 2006 ▸; Deng *et al.*, 2016 ▸; Peng *et al.*, 2017 ▸). Remarkable chelating ability together with synthetic accessibility led to the exploration of aroylhydrazones as potential metal-chelating drugs (Link *et al.*, 2003 ▸; Bernhardt *et al.*, 2007 ▸). Another field of application includes utilization of some aroylhydrazones as fluorescent probes and as metal-ion fluorescence chemosensors (Xiang *et al.*, 2006 ▸; Wu *et al.*, 2007 ▸).

The aroylhydrazone ligands can form charged complexes or can easily be deprotonated due to tautomerism, thus forming neutral species. These dynamic reversible properties have led to the exploration of charged and neutral spin-crossover iron(II) and iron(III) complexes, some with multifunctional properties (Zhang *et al.*, 2010 ▸; Shongwe *et al.*, 2012 ▸; Romero-Morcillo *et al.*, 2015 ▸; Yuan *et al.*, 2019 ▸). As part of our contin­uing inter­est in studying 3*d* metal complexes formed by polydentate ligands bearing alk­oxy substituents (Seredyuk, 2012 ▸; Seredyuk *et al.*, 2006 ▸, 2011 ▸, 2016 ▸) and those based on polydentate ligands (Seredyuk *et al.*, 2007 ▸, 2015 ▸), we report here the synthesis and crystal structure of a neutral Zn^II^ complex formed with the tridentate ligand 3,4,5-trimeth­oxy-*N*′-[1-(pyridin-2-yl)ethyl­idene]benzohydrazide.
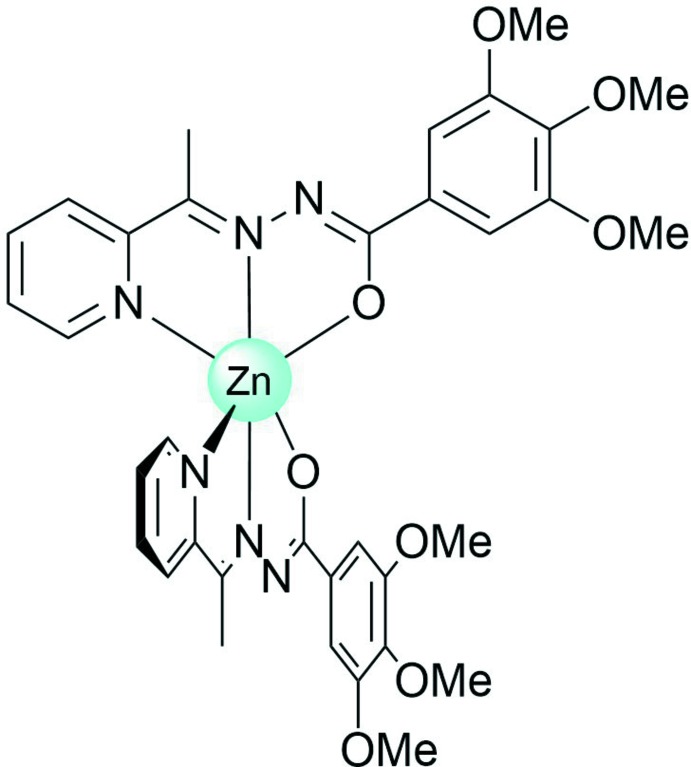


## Structural commentary   

**Figure 1:**
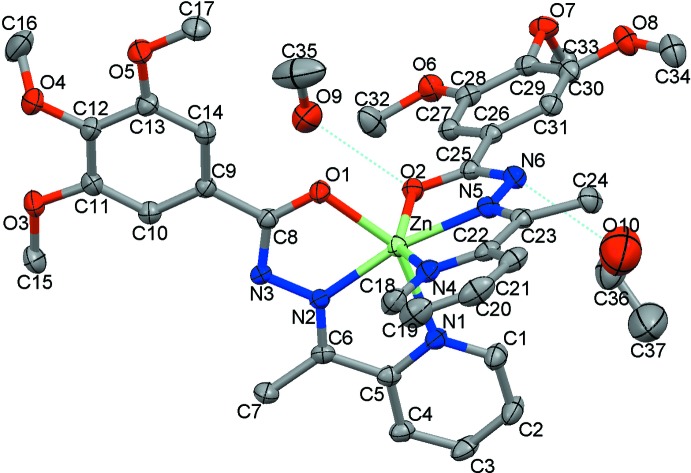
The title compound with the atom-numbering scheme. Displacement ellipsoids are drawn at the 50% probability level. H atoms have been omitted for clarity.

In the complex, the Zn^II^ ion possesses a distorted octa­hedral N_4_O_2_ coordination environment, which is generated by the two deprotonated ligands (Fig. 1 ▸). The average bond lengths [Zn—N = 2.145 (3) Å and Zn—O = 2.141 (2) Å] are typical for such Zn^II^ complexes (Jang *et al.*, 2005 ▸; Barbazán *et al.*, 2007 ▸; Singh *et al.*, 2015 ▸; Kane *et al.*, 2016 ▸; Wang *et al.*, 2019 ▸). The N2—Zn—N5 angle, formed by the ketimine N atoms of the two ligand mol­ecules, is 164.81 (10)°, showing the deviation of the coordination polyhedron from an ideal octa­hedral geometry. The average trigonal distortion parameters Σ = Σ_1_^24^(60 − θ_i_)/24, where θ_i_ is the angle generated by superposition of two opposite faces of the octa­hedron (Chang *et al.*, 1990 ▸) and Φ = Σ_1_^12^(|φ_i_ − 90|)/12, where φ_i_ is the deviation from 90° of the *cis*-N—Zn—N angles in the coordination sphere (Drew *et al.*, 1995 ▸), are 18.38 and 11.65°, respectively, which correspond to a moderate distortion. The volume of the coordination polyhedron is 12.008 Å^3^.

## Supra­molecular features   

**Figure 2:**
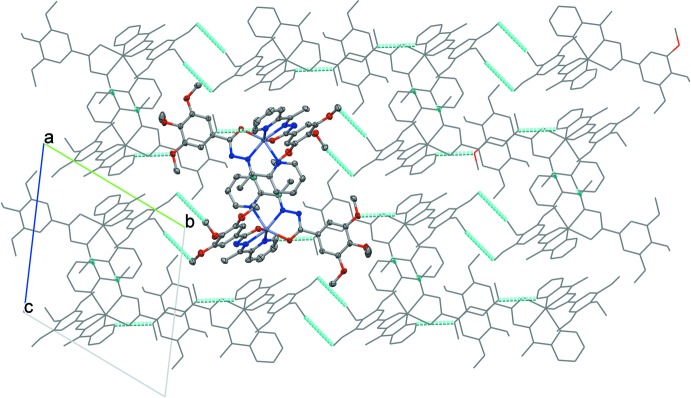
The packing of mol­ecules, showing as dashed lines the inter­actions below the sum of the van der Waals radii. The supra­molecular dimer is also highlighted.

The ligand mol­ecules exhibit slipped parallel π–π stacking between coplanar ligands of neighbouring mol­ecules, thus forming a dimeric structure; the closest C4⋯C6^i^/C6⋯C4^i^ contacts, below the sum of the van der Waals radii, are 3.374 (5) Å. In the dimer, the Zn⋯Zn^i^ separation is 7.612 (2) Å [symmetry code: (i) −*x*, −*y* + 1, −*z* + 1] (Fig. 2 ▸). Neighbouring dimers are bound along [010] by weak hydrogen bonds between the pyridine rings and meth­oxy groups, C18⋯O3^ii^ [symmetry code: (ii) −*x*, −*y*, −*z* + 1] = 3.100 (5) Å (Table 1 ▸), with the closest Zn⋯Zn^ii^ inter­dimer separation of 6.965 (5) Å. It is worth noting that a related Fe^II^ pyridine-based complex with butyl substituents consisting of uniform supra­molecular chains with Fe⋯Fe separation of 7.676 Å has previously been described (Romero-Morcillo *et al.*, 2015 ▸). The supra­molecular chains of the title compound are packed in the lattice with the closest inter­chain separations coinciding with the unit-cell parameters *a* = 11.0402 (4) Å and *b* = 13.8056 (8) Å. There are inter­chain contacts C33⋯C34^iii^/C34⋯C33^iii^ [symmetry code: (iii) −*x* + 1, −*y* + 2, −*z*], below the sum of the van der Waals radii, between the meth­oxy groups of neighbouring supra­molecular chains at 3.385 (5) Å.

## Co-crystallized methanol and ethanol   

The neutral nature of the complex mol­ecule and therefore the absence of anions and, on the other hand, the relatively large size of the planar rigid substituents prevent the formation of a tightly packed lattice. Therefore, inter­molecular voids are filled by the co-crystallized mol­ecules of ethanol, which act as bridges connecting the closest complex mol­ecules by O—H⋯N hydrogen bonding, with the distance between the donor and acceptor atoms O10⋯N6 equal to 2.825 (5) Å. The contact C15⋯C37^iv^ [symmetry code: (iv) −*x*, −*y* + 1, −*z* + 1] between the ethanol methyl group and a meth­oxy methyl group is 3.300 (5)Å. Additionally, neighbouring mol­ecules of ethanol are mutually bound forming dimers with C36⋯C37^v^ and O10⋯C37^v^ [symmetry code: (v) −*x*, −*y* + 2, −*z*] contacts with distances of 3.227 (5) and 2.751 (2) Å, respectively. Furthermore, the co-crystallized mol­ecules of methanol form O—H⋯O hydrogen bonds with the meth­oxy group of the ligand, with an O9⋯O2 separation between the O atoms of 2.776 (4) Å.

## Hirshfeld surface and 2D fingerprint plots   

**Figure 3:**
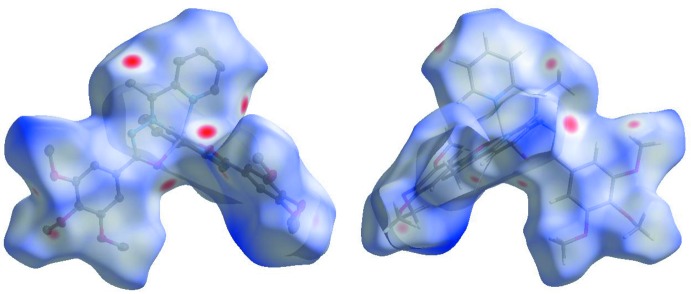
Two projections of *d*_norm_ mapped on Hirshfeld surfaces, showing the inter­molecular inter­actions within the mol­ecule. Red areas represent contacts shorter than the sum of the van der Waals radii, while blue areas represent regions where contacts are larger than the sum of van der Waals radii, and white areas are zones close to the sum of van der Waals radii.

**Figure 4:**
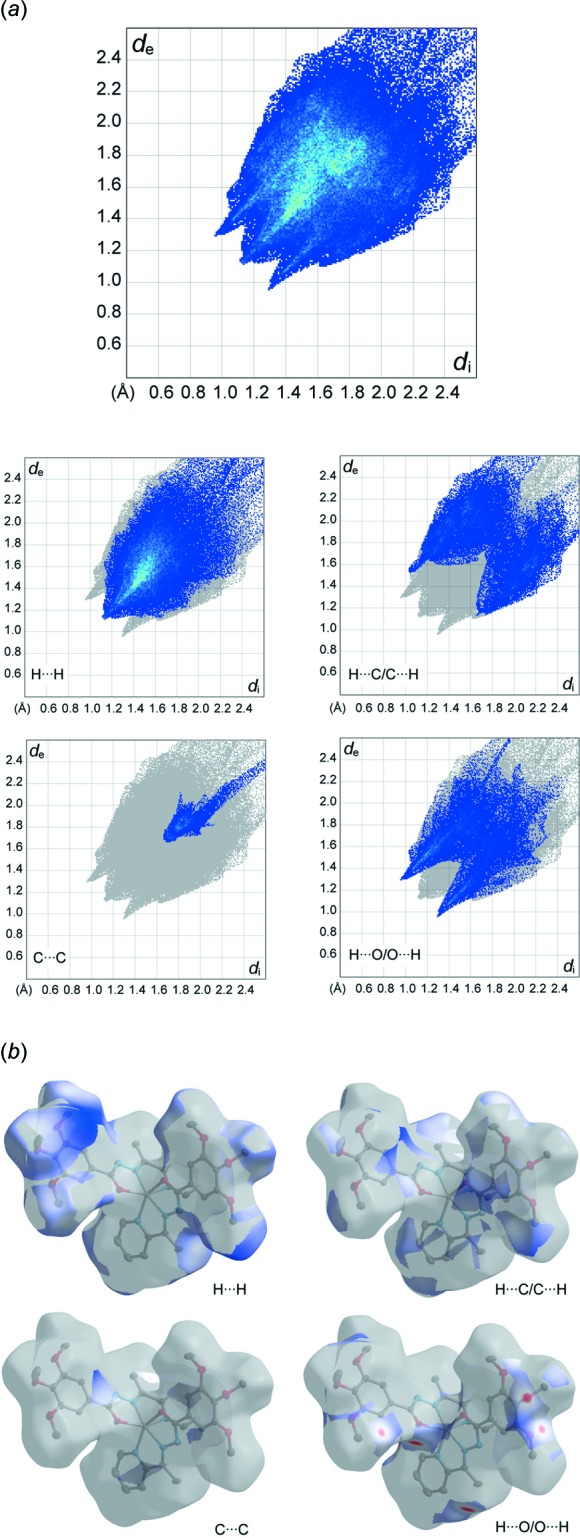
(*a*) The overall two-dimensional fingerprint plot and those decomposed into specified inter­actions. (*b*) Hirshfeld surface representations with the function *d*_norm_ plotted onto the surface for the different inter­actions.

The Hirshfeld surface analysis and the associated two-dimensional fingerprint plots were undertaken using *CrystalExplorer17.5* software (Turner *et al.*, 2018 ▸), using standard surface resolution with the three-dimensional *d*_norm_ surfaces plotted over a fixed colour scale of −0.2580 (red) to 2.2951 (blue) a.u. The pale-red spots symbolize short contacts and negative *d*_norm_ values on the surface correspond to the inter­actions described above. The overall two-dimensional fingerprint plot is illustrated in Fig. 3 ▸. The Hirshfeld surfaces mapped over *d*_norm_ are shown for the H⋯H, H⋯C/C⋯H, H⋯O/O⋯H, and C⋯C contacts, and the two-dimensional fingerprint plots are presented in Fig. 4 ▸, associated with their relative contributions to the Hirshfeld surface. At 44.8%, the largest contribution to the overall crystal packing is from H⋯H inter­actions, which are located in the middle region of the fingerprint plot. H⋯C/C⋯H contacts contribute to 22.2% to the Hirshfeld surface, resulting in two pairs of characteristic wings. The pair of tips of H⋯O/O⋯H contacts make a 18.7% contribution to the Hirshfeld surface. The contacts are represented by a pair of sharp spikes in the fingerprint plot. The C⋯C contacts contribute only to 3.9% to the Hirshfeld surface.

## Database survey   

A search of the Cambridge Structural Database (CSD, Version 5.40, update November 2018; Groom *et al.*, 2016 ▸) revealed four structurally similar Zn complexes based on ligands without or with substituents on the phenyl ring: *N*′-[1-(pyridin-2-yl)ethyl­idene]benzohydrazide (PATXAK; Jang *et al.*, 2005 ▸), 2-amino-*N*′-[1-(pyridin-2-yl)ethyl­idene]benzohydrazide (MAKLES; Kane *et al.*, 2016 ▸), 2-hy­droxy-*N*′-[1-(pyridin-2-yl)ethyl­idene]benzohydrazide (HIGPOD; Barbazán *et al.*, 2007 ▸) and 3-methyl-*N*′-[1-(pyridin-2-yl)ethyl­idene]benzohydrazide (POKPAJ; Wang *et al.*, 2019 ▸). PATXAK crystallizes in the space group *C*2/*c*, both MAKLES and POKPAJ in *P*2_1_/*c* and HIGPOD in *Aba*2. The N—Zn—N angle, formed by the apical ketimine N atoms and the central Zn atom, varies from 163.05 (POKPAJ) to 177.76° (MAKLES), while inter­mediate values of 168.09 and 170.56° are observed for PATXAK and HIGPOD, respectively.

## Synthesis and crystallization   

The complex was obtained by condensation of 3,4,5-tri­meth­oxy­benzohydrazide (1 mmol) and acetyl pyridine (1.1 mmol) in a mixture of absolute MeOH and EtOH (1:1) overnight in the presence of two drops of glacial acetic acid. The ligand obtained *in situ* was subsequently reacted with solid ZnCl_2_·6H_2_O (0.5 mmol) to give a colourless complex. A pale-yellow solution was obtained after deprotonation with NEt_3_ (1 mmol). The neutral complex was isolated by slow cooling the solution to ambient temperature and subsequently by filtering off the yellowish crystals. Elemental analysis calculated (%) for C_37_H_46_N_6_O_10_Zn: C 55.54, H 5.79, N 10.50; found: C 55.86, H 5.31, N 10.84. IR νKBr (cm^−1^): 1617 (N=C—O), 1588, 1461 (C=N^py^ + C=C^Ar^), 1252 (C—O). MS ESI *m*/*z* (relative intensity): theoretically calculated 721.19 [*M* + H^+^] (100.0%). Found 721.21 [*M* + H^+^] (100.0%). TGA (up to 400 K) expected weight loss for EtOH + MeOH: 9.8%; found: 9.5%.

## Refinement   

Crystal data, data collection and structure refinement details are summarized in Table 2 ▸. H atoms were placed in calculated positions using idealized geometries, with C—H = 0.97 Å for mthyl goups and 0.93 Å for aromatic H atoms, and refined using a riding model with *U*_iso_(H) = 1.2–1.5*U*_eq_(C). None of the hydrogen atoms of the methanol or ethanol molecules could be located.

## Supplementary Material

Crystal structure: contains datablock(s) I. DOI: 10.1107/S2056989020000857/tx2017sup1.cif

Structure factors: contains datablock(s) I. DOI: 10.1107/S2056989020000857/tx2017Isup2.hkl

Supporting information file. DOI: 10.1107/S2056989020000857/tx2017Isup3.cdx

CCDC reference: 1979477

Additional supporting information:  crystallographic information; 3D view; checkCIF report

## Figures and Tables

**Table 1:** Hydrogen-bond geometry (Å, °)

*D*—H⋯*A*	*D*—H	H⋯*A*	*D*⋯*A*	*D*—H⋯*A*
C16—H16*C*⋯O5	0.96	2.58	3.097 (7)	114
C17—H17*B*⋯O8^i^	0.96	2.59	3.457 (6)	150
C18—H18⋯O3^ii^	0.93	2.42	3.100 (5)	130
C24—H24*B*⋯O7^iii^	0.96	2.55	3.414 (5)	149
C24—H24*C*⋯O1^iv^	0.96	2.38	3.281 (4)	157
C33—H33*B*⋯O6	0.96	2.54	3.075 (5)	115

Symmetry codes: (i) 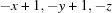; (ii) 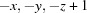; (iii) 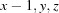; (iv) 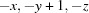.

**Table 2:** Experimental details

Crystal data	
Chemical formula	[Zn(C_17_H_18_N_3_O_4_)_2_]·CH_4_O·C_2_H_6_O
*M*_r_	790.11
Crystal system, space group	Triclinic, *P* 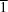
Temperature (K)	120
*a*, *b*, *c* (Å)	11.0402 (4), 13.8056 (8), 14.4190 (7)
α, β, γ (°)	63.256 (5), 74.098 (4), 75.307 (4)
*V* (Å^3^)	1865.63 (18)
*Z*	2
Radiation type	Mo *K*α
μ (mm^−1^)	0.72
Crystal size (mm)	0.09 × 0.02 × 0.02

Data collection	
Diffractometer	Agilent SuperNova Sapphire3
Absorption correction	Multi-scan (*CrysAlis PRO*; Agilent, 2012 ▸)
*T*_min_, *T*_max_	0.768, 1.000
No. of measured, independent and observed [*I* > 2σ(*I*)] reflections	18302, 9361, 7343
*R*_int_	0.040
(sin θ/λ)_max_ (Å^−1^)	0.701

Refinement	
*R*[*F* ^2^ > 2σ(*F* ^2^)], *wR*(*F* ^2^), *S*	0.064, 0.192, 0.90
No. of reflections	9361
No. of parameters	487
H-atom treatment	H-atom parameters constrained
Δρ_max_, Δρ_min_ (e Å^−3^)	2.11, −0.73

Computer programs: *CrysAlis PRO* (Agilent, 2012 ▸), *SHELXS2014* (Sheldrick, 2015*a* ▸) and *SHELXL2014* (Sheldrick, 2015*b* ▸).

## supplementary crystallographic information

### Crystal data

**Table:**

[Zn(C_17_H_18_N_3_O_4_)_2_]·CH_4_O·C_2_H_6_O	*Z* = 2
*M**_r_* = 790.11	*F*(000) = 836
Triclinic, *P*1	*D*_x_ = 1.421 Mg m^−^^3^
*a* = 11.0402 (4) Å	Mo *K*α radiation, λ = 0.71069 Å
*b* = 13.8056 (8) Å	Cell parameters from 5835 reflections
*c* = 14.4190 (7) Å	θ = 4.7–20.1°
α = 63.256 (5)°	µ = 0.72 mm^−^^1^
β = 74.098 (4)°	*T* = 120 K
γ = 75.307 (4)°	Prismatic, yellow
*V* = 1865.63 (18) Å^3^	0.09 × 0.02 × 0.02 mm

### Data collection

**Table:**

Agilent SuperNova Sapphire3 diffractometer	7343 reflections with *I* > 2σ(*I*)
φ scans and ω scans with κ offset	*R*_int_ = 0.040
Absorption correction: multi-scan (CrysAlis PRO; Agilent, 2012)	θ_max_ = 29.9°, θ_min_ = 3.0°
*T*_min_ = 0.768, *T*_max_ = 1.000	*h* = −14→15
18302 measured reflections	*k* = −19→18
9361 independent reflections	*l* = −19→20

### Refinement

**Table:**

Refinement on *F*^2^	0 constraints
Least-squares matrix: full	Hydrogen site location: inferred from neighbouring sites
*R*[*F*^2^ > 2σ(*F*^2^)] = 0.064	H-atom parameters constrained
*wR*(*F*^2^) = 0.192	*w* = 1/[σ^2^(*F*_o_^2^) + (0.1156*P*)^2^ + 3.6309*P*] where *P* = (*F*_o_^2^ + 2*F*_c_^2^)/3
*S* = 0.90	(Δ/σ)_max_ < 0.001
9361 reflections	Δρ_max_ = 2.11 e Å^−^^3^
487 parameters	Δρ_min_ = −0.73 e Å^−^^3^
0 restraints	

### Special details

**Table:**

Experimental. CrysAlisPro, Agilent Technologies, Version 1.171.36.21 (release 14-08-2012 CrysAlis171 .NET) (compiled Sep 14 2012,17:21:16) Empirical absorption correction using spherical harmonics, implemented in SCALE3 ABSPACK scaling algorithm.
Geometry. All esds (except the esd in the dihedral angle between two l.s. planes) are estimated using the full covariance matrix. The cell esds are taken into account individually in the estimation of esds in distances, angles and torsion angles; correlations between esds in cell parameters are only used when they are defined by crystal symmetry. An approximate (isotropic) treatment of cell esds is used for estimating esds involving l.s. planes.

### Fractional atomic coordinates and isotropic or equivalent isotropic displacement parameters (Å^2^)

**Table:**

	*x*	*y*	*z*	*U*_iso_*/*U*_eq_	
Zn	0.00210 (3)	0.45634 (3)	0.25703 (3)	0.02225 (12)	
N1	−0.1206 (2)	0.5723 (2)	0.3324 (2)	0.0240 (5)	
N2	−0.0307 (2)	0.3641 (2)	0.4169 (2)	0.0211 (5)	
N3	0.0174 (2)	0.2545 (2)	0.4518 (2)	0.0224 (5)	
N4	−0.1512 (3)	0.4591 (2)	0.1888 (2)	0.0260 (5)	
N5	0.0250 (2)	0.5836 (2)	0.1077 (2)	0.0224 (5)	
N6	0.1200 (3)	0.6457 (2)	0.0775 (2)	0.0250 (5)	
O1	0.0860 (2)	0.29614 (18)	0.27196 (17)	0.0255 (5)	
O2	0.1803 (2)	0.51141 (18)	0.23406 (17)	0.0244 (4)	
O3	0.2036 (3)	−0.1382 (2)	0.6353 (2)	0.0374 (6)	
O4	0.2942 (3)	−0.2172 (2)	0.4875 (2)	0.0384 (6)	
O5	0.2874 (3)	−0.0886 (2)	0.2824 (2)	0.0368 (6)	
O6	0.5751 (2)	0.6309 (2)	0.2440 (2)	0.0324 (5)	
O7	0.6340 (2)	0.7917 (2)	0.0516 (2)	0.0314 (5)	
O8	0.4815 (3)	0.8724 (2)	−0.0894 (2)	0.0352 (6)	
C1	−0.1619 (4)	0.6792 (3)	0.2871 (3)	0.0320 (7)	
H1	−0.1502	0.7130	0.2137	0.038*	
C2	−0.2216 (4)	0.7429 (3)	0.3436 (3)	0.0360 (8)	
H2	−0.2483	0.8177	0.3088	0.043*	
C3	−0.2405 (3)	0.6932 (3)	0.4520 (3)	0.0333 (8)	
H3	−0.2795	0.7341	0.4918	0.040*	
C4	−0.2005 (3)	0.5812 (3)	0.5015 (3)	0.0264 (6)	
H4	−0.2133	0.5458	0.5748	0.032*	
C5	−0.1408 (3)	0.5225 (3)	0.4396 (2)	0.0224 (6)	
C6	−0.0939 (3)	0.4027 (3)	0.4855 (2)	0.0211 (6)	
C7	−0.1203 (3)	0.3345 (3)	0.6013 (3)	0.0293 (7)	
H7A	−0.0826	0.2597	0.6153	0.035*	
H7B	−0.2107	0.3384	0.6262	0.035*	
H7C	−0.0847	0.3616	0.6372	0.035*	
C8	0.0760 (3)	0.2301 (2)	0.3693 (2)	0.0215 (6)	
C9	0.1334 (3)	0.1117 (2)	0.3994 (2)	0.0215 (6)	
C10	0.1370 (3)	0.0439 (3)	0.5047 (3)	0.0256 (6)	
H10	0.1023	0.0717	0.5559	0.031*	
C11	0.1919 (3)	−0.0652 (3)	0.5340 (3)	0.0273 (6)	
C12	0.2428 (3)	−0.1075 (3)	0.4567 (3)	0.0280 (7)	
C13	0.2372 (3)	−0.0390 (3)	0.3511 (3)	0.0276 (7)	
C14	0.1839 (3)	0.0712 (3)	0.3217 (3)	0.0246 (6)	
H14	0.1820	0.1172	0.2510	0.030*	
C15	0.1521 (4)	−0.0971 (3)	0.7155 (3)	0.0363 (8)	
H15A	0.1653	−0.1546	0.7830	0.044*	
H15B	0.0625	−0.0720	0.7168	0.044*	
H15C	0.1940	−0.0373	0.7005	0.044*	
C16	0.4298 (5)	−0.2361 (4)	0.4685 (5)	0.0631 (15)	
H16A	0.4595	−0.3135	0.4919	0.076*	
H16B	0.4596	−0.2076	0.5066	0.076*	
H16C	0.4617	−0.1998	0.3943	0.076*	
C17	0.2735 (4)	−0.0242 (3)	0.1750 (3)	0.0362 (8)	
H17A	0.3123	−0.0669	0.1343	0.043*	
H17B	0.3143	0.0396	0.1471	0.043*	
H17C	0.1846	−0.0017	0.1713	0.043*	
C18	−0.2358 (3)	0.3898 (3)	0.2326 (3)	0.0351 (8)	
H18	−0.2334	0.3359	0.3007	0.042*	
C19	−0.3270 (4)	0.3949 (3)	0.1806 (4)	0.0431 (10)	
H19	−0.3851	0.3456	0.2130	0.052*	
C20	−0.3301 (4)	0.4750 (4)	0.0795 (4)	0.0429 (10)	
H20	−0.3900	0.4797	0.0426	0.051*	
C21	−0.2432 (3)	0.5486 (3)	0.0331 (3)	0.0335 (8)	
H21	−0.2441	0.6031	−0.0349	0.040*	
C22	−0.1551 (3)	0.5390 (3)	0.0903 (3)	0.0248 (6)	
C23	−0.0583 (3)	0.6128 (3)	0.0487 (2)	0.0238 (6)	
C24	−0.0616 (3)	0.7115 (3)	−0.0537 (3)	0.0309 (7)	
H24A	0.0076	0.7497	−0.0685	0.037*	
H24B	−0.1410	0.7592	−0.0489	0.037*	
H24C	−0.0536	0.6890	−0.1094	0.037*	
C25	0.1960 (3)	0.5981 (2)	0.1491 (2)	0.0213 (6)	
C26	0.3095 (3)	0.6523 (3)	0.1235 (2)	0.0226 (6)	
C27	0.3861 (3)	0.6143 (3)	0.1989 (3)	0.0243 (6)	
H27	0.3661	0.5569	0.2641	0.029*	
C28	0.4926 (3)	0.6627 (3)	0.1762 (3)	0.0260 (6)	
C29	0.5235 (3)	0.7488 (3)	0.0781 (3)	0.0261 (6)	
C30	0.4442 (3)	0.7873 (3)	0.0029 (3)	0.0274 (7)	
C31	0.3379 (3)	0.7390 (3)	0.0254 (3)	0.0260 (6)	
H31	0.2859	0.7641	−0.0243	0.031*	
C32	0.5417 (4)	0.5483 (4)	0.3476 (3)	0.0409 (9)	
H32A	0.6061	0.5323	0.3878	0.049*	
H32B	0.5352	0.4830	0.3423	0.049*	
H32C	0.4613	0.5743	0.3821	0.049*	
C33	0.6268 (3)	0.8663 (3)	0.0971 (3)	0.0313 (7)	
H33A	0.7066	0.8930	0.0755	0.038*	
H33B	0.6085	0.8292	0.1728	0.038*	
H33C	0.5603	0.9269	0.0738	0.038*	
C34	0.3957 (4)	0.9227 (3)	−0.1632 (3)	0.0412 (9)	
H34A	0.4313	0.9813	−0.2248	0.049*	
H34B	0.3155	0.9514	−0.1308	0.049*	
H34C	0.3830	0.8690	−0.1832	0.049*	
C35	0.3927 (6)	0.2790 (6)	0.2348 (7)	0.085 (2)	
C36	0.0330 (5)	0.8853 (4)	0.1189 (5)	0.0566 (12)	
O10	−0.0012 (6)	0.8601 (4)	0.0529 (5)	0.1041 (17)	
O9	0.3710 (3)	0.3347 (3)	0.3011 (3)	0.0568 (8)	
C37	−0.0639 (9)	0.9623 (6)	0.1313 (7)	0.109 (3)	

### Atomic displacement parameters (Å^2^)

**Table:**

	*U*^11^	*U*^22^	*U*^33^	*U*^12^	*U*^13^	*U*^23^
Zn	0.0247 (2)	0.02143 (19)	0.01899 (19)	−0.00006 (13)	−0.00520 (13)	−0.00800 (14)
N1	0.0235 (12)	0.0243 (13)	0.0246 (13)	0.0014 (10)	−0.0062 (10)	−0.0118 (11)
N2	0.0193 (11)	0.0221 (12)	0.0204 (12)	0.0003 (9)	−0.0037 (9)	−0.0093 (10)
N3	0.0212 (12)	0.0203 (12)	0.0218 (12)	0.0005 (10)	−0.0015 (9)	−0.0086 (10)
N4	0.0262 (13)	0.0233 (12)	0.0296 (14)	−0.0014 (10)	−0.0051 (11)	−0.0131 (11)
N5	0.0220 (12)	0.0267 (13)	0.0196 (12)	−0.0025 (10)	−0.0048 (9)	−0.0104 (11)
N6	0.0251 (13)	0.0262 (13)	0.0225 (13)	−0.0055 (10)	−0.0044 (10)	−0.0081 (11)
O1	0.0325 (12)	0.0212 (10)	0.0194 (10)	0.0023 (9)	−0.0060 (9)	−0.0080 (9)
O2	0.0245 (11)	0.0251 (11)	0.0210 (10)	−0.0025 (9)	−0.0057 (8)	−0.0071 (9)
O3	0.0494 (15)	0.0250 (12)	0.0233 (12)	0.0014 (11)	−0.0034 (11)	−0.0031 (10)
O4	0.0468 (15)	0.0206 (11)	0.0363 (14)	0.0005 (10)	−0.0006 (12)	−0.0086 (11)
O5	0.0500 (15)	0.0259 (12)	0.0300 (13)	0.0016 (11)	−0.0004 (11)	−0.0152 (11)
O6	0.0263 (11)	0.0366 (13)	0.0325 (13)	−0.0071 (10)	−0.0101 (10)	−0.0085 (11)
O7	0.0249 (11)	0.0335 (12)	0.0404 (14)	−0.0089 (10)	0.0009 (10)	−0.0206 (11)
O8	0.0454 (15)	0.0297 (12)	0.0293 (13)	−0.0155 (11)	−0.0050 (11)	−0.0072 (11)
C1	0.0395 (18)	0.0274 (16)	0.0268 (16)	0.0049 (14)	−0.0116 (14)	−0.0111 (14)
C2	0.047 (2)	0.0244 (16)	0.0361 (19)	0.0095 (15)	−0.0166 (16)	−0.0142 (15)
C3	0.0341 (18)	0.0354 (18)	0.0371 (19)	0.0090 (14)	−0.0118 (15)	−0.0250 (16)
C4	0.0248 (15)	0.0310 (16)	0.0253 (15)	0.0009 (12)	−0.0049 (12)	−0.0157 (14)
C5	0.0191 (13)	0.0274 (15)	0.0230 (14)	−0.0023 (11)	−0.0044 (11)	−0.0125 (12)
C6	0.0175 (13)	0.0250 (14)	0.0232 (14)	−0.0033 (11)	−0.0018 (11)	−0.0130 (12)
C7	0.0319 (17)	0.0303 (16)	0.0224 (15)	−0.0013 (13)	−0.0008 (12)	−0.0121 (13)
C8	0.0207 (13)	0.0215 (13)	0.0222 (14)	−0.0011 (11)	−0.0053 (11)	−0.0091 (12)
C9	0.0195 (13)	0.0202 (13)	0.0239 (14)	−0.0035 (11)	−0.0030 (11)	−0.0085 (12)
C10	0.0260 (15)	0.0247 (15)	0.0241 (15)	−0.0024 (12)	−0.0035 (12)	−0.0098 (13)
C11	0.0281 (15)	0.0240 (15)	0.0233 (15)	−0.0051 (12)	−0.0014 (12)	−0.0054 (13)
C12	0.0290 (16)	0.0192 (14)	0.0300 (16)	−0.0020 (12)	−0.0008 (13)	−0.0086 (13)
C13	0.0300 (16)	0.0232 (15)	0.0297 (16)	−0.0042 (12)	−0.0014 (13)	−0.0129 (13)
C14	0.0273 (15)	0.0208 (14)	0.0233 (14)	−0.0040 (12)	−0.0039 (12)	−0.0072 (12)
C15	0.043 (2)	0.0354 (18)	0.0214 (16)	−0.0015 (15)	−0.0073 (14)	−0.0051 (14)
C16	0.046 (3)	0.033 (2)	0.082 (4)	0.0092 (19)	−0.002 (2)	−0.014 (2)
C17	0.042 (2)	0.0381 (19)	0.0288 (17)	−0.0025 (16)	−0.0030 (15)	−0.0183 (16)
C18	0.0287 (17)	0.0268 (16)	0.047 (2)	−0.0019 (13)	−0.0064 (15)	−0.0142 (16)
C19	0.0298 (18)	0.039 (2)	0.069 (3)	−0.0082 (16)	−0.0088 (18)	−0.028 (2)
C20	0.0281 (17)	0.050 (2)	0.068 (3)	0.0022 (16)	−0.0181 (18)	−0.039 (2)
C21	0.0303 (17)	0.0400 (19)	0.0384 (19)	0.0067 (14)	−0.0134 (14)	−0.0254 (17)
C22	0.0214 (14)	0.0273 (15)	0.0300 (16)	0.0018 (12)	−0.0044 (12)	−0.0185 (13)
C23	0.0259 (15)	0.0280 (15)	0.0186 (14)	0.0005 (12)	−0.0040 (11)	−0.0128 (12)
C24	0.0309 (16)	0.0376 (18)	0.0206 (15)	−0.0027 (14)	−0.0086 (12)	−0.0079 (14)
C25	0.0206 (13)	0.0228 (14)	0.0206 (13)	−0.0017 (11)	−0.0019 (11)	−0.0109 (12)
C26	0.0236 (14)	0.0230 (14)	0.0218 (14)	−0.0009 (11)	−0.0032 (11)	−0.0118 (12)
C27	0.0225 (14)	0.0259 (15)	0.0240 (15)	−0.0020 (12)	−0.0033 (11)	−0.0111 (13)
C28	0.0224 (14)	0.0290 (16)	0.0291 (16)	−0.0009 (12)	−0.0051 (12)	−0.0156 (14)
C29	0.0223 (14)	0.0276 (15)	0.0306 (16)	−0.0035 (12)	−0.0003 (12)	−0.0166 (14)
C30	0.0341 (17)	0.0227 (14)	0.0248 (15)	−0.0062 (13)	−0.0009 (13)	−0.0109 (13)
C31	0.0294 (15)	0.0256 (15)	0.0226 (15)	−0.0029 (12)	−0.0052 (12)	−0.0101 (13)
C32	0.0336 (18)	0.056 (2)	0.0279 (18)	−0.0115 (17)	−0.0112 (14)	−0.0069 (17)
C33	0.0333 (17)	0.0299 (17)	0.0351 (18)	−0.0082 (14)	−0.0087 (14)	−0.0140 (15)
C34	0.057 (2)	0.0334 (19)	0.0326 (19)	−0.0132 (18)	−0.0104 (17)	−0.0085 (16)
C35	0.055 (3)	0.091 (4)	0.136 (6)	−0.028 (3)	0.025 (3)	−0.084 (5)
C36	0.071 (3)	0.039 (2)	0.066 (3)	−0.006 (2)	−0.019 (3)	−0.024 (2)
O10	0.120 (4)	0.081 (3)	0.104 (4)	−0.006 (3)	−0.028 (3)	−0.032 (3)
O9	0.0526 (19)	0.0516 (19)	0.0488 (19)	0.0089 (15)	−0.0077 (15)	−0.0154 (16)
C37	0.132 (7)	0.074 (4)	0.080 (5)	0.018 (4)	0.003 (5)	−0.027 (4)

### Geometric parameters (Å, º)

**Table:**

Zn—O1	2.106 (2)	C1—C2	1.390 (5)
Zn—O2	2.176 (2)	C2—C3	1.373 (5)
Zn—N1	2.289 (3)	C3—C4	1.389 (5)
Zn—N2	2.049 (3)	C4—C5	1.395 (4)
Zn—N4	2.164 (3)	C5—C6	1.486 (4)
Zn—N5	2.076 (3)	C6—C7	1.489 (4)
N1—C1	1.328 (4)	C8—C9	1.500 (4)
N1—C5	1.358 (4)	C9—C14	1.393 (4)
N2—C6	1.288 (4)	C9—C10	1.385 (4)
N2—N3	1.370 (4)	C10—C11	1.385 (4)
N3—C8	1.333 (4)	C11—C12	1.407 (5)
N4—C18	1.331 (5)	C12—C13	1.393 (5)
N4—C22	1.354 (4)	C13—C14	1.394 (4)
N5—C23	1.290 (4)	C18—C19	1.383 (5)
N5—N6	1.377 (4)	C19—C20	1.380 (7)
N6—C25	1.336 (4)	C20—C21	1.390 (6)
O1—C8	1.276 (4)	C21—C22	1.385 (4)
O2—C25	1.278 (4)	C22—C23	1.480 (5)
O3—C11	1.370 (4)	C23—C24	1.494 (5)
O3—C15	1.431 (4)	C25—C26	1.497 (4)
O4—C12	1.382 (4)	C26—C31	1.398 (4)
O4—C16	1.423 (6)	C26—C27	1.393 (4)
O5—C13	1.371 (4)	C27—C28	1.387 (4)
O5—C17	1.426 (4)	C28—C29	1.400 (5)
O6—C28	1.369 (4)	C29—C30	1.410 (5)
O6—C32	1.430 (5)	C30—C31	1.386 (5)
O7—C29	1.377 (4)	C35—O9	1.416 (7)
O7—C33	1.427 (4)	C36—O10	1.315 (7)
O8—C30	1.364 (4)	C36—C37	1.339 (9)
O8—C34	1.437 (5)		
			
O1—Zn—O2	95.27 (9)	C7—C6—C5	121.9 (3)
O1—Zn—N1	149.55 (9)	O1—C8—N3	126.6 (3)
N2—Zn—O1	76.21 (9)	O1—C8—C9	119.7 (3)
O1—Zn—N4	93.40 (9)	N3—C8—C9	113.7 (3)
N5—Zn—O1	118.14 (9)	C14—C9—C10	120.7 (3)
N2—Zn—O2	101.58 (9)	C14—C9—C8	120.1 (3)
N4—Zn—O2	148.23 (10)	C10—C9—C8	119.2 (3)
N5—Zn—O2	73.41 (9)	C9—C10—C11	120.3 (3)
N2—Zn—N1	73.66 (10)	O3—C11—C10	124.9 (3)
N4—Zn—N1	92.84 (10)	O3—C11—C12	115.3 (3)
N5—Zn—N1	92.26 (10)	C10—C11—C12	119.8 (3)
N2—Zn—N4	110.17 (11)	O4—C12—C13	121.4 (3)
N2—Zn—N5	164.81 (10)	O4—C12—C11	119.1 (3)
N5—Zn—N4	75.54 (10)	C13—C12—C11	119.4 (3)
O2—Zn—N1	94.93 (9)	O5—C13—C12	114.8 (3)
C1—N1—C5	118.2 (3)	O5—C13—C14	124.6 (3)
C1—N1—Zn	129.5 (2)	C12—C13—C14	120.6 (3)
C5—N1—Zn	112.0 (2)	C9—C14—C13	119.2 (3)
C6—N2—N3	118.6 (3)	N4—C18—C19	122.4 (4)
C6—N2—Zn	124.0 (2)	C20—C19—C18	118.6 (4)
N3—N2—Zn	117.41 (19)	C19—C20—C21	119.7 (3)
C8—N3—N2	109.5 (2)	C22—C21—C20	118.5 (4)
C18—N4—C22	119.4 (3)	N4—C22—C21	121.5 (3)
C18—N4—Zn	126.6 (3)	N4—C22—C23	115.4 (3)
C22—N4—Zn	114.0 (2)	C21—C22—C23	123.1 (3)
C23—N5—N6	119.8 (3)	N5—C23—C22	114.3 (3)
C23—N5—Zn	120.1 (2)	N5—C23—C24	125.1 (3)
N6—N5—Zn	119.52 (19)	C22—C23—C24	120.6 (3)
C25—N6—N5	109.0 (3)	O2—C25—N6	125.6 (3)
C8—O1—Zn	110.20 (19)	O2—C25—C26	119.0 (3)
C25—O2—Zn	111.36 (18)	N6—C25—C26	115.3 (3)
C11—O3—C15	116.3 (3)	C31—C26—C27	120.9 (3)
C12—O4—C16	113.5 (3)	C31—C26—C25	120.4 (3)
C13—O5—C17	116.7 (3)	C27—C26—C25	118.7 (3)
C28—O6—C32	116.7 (3)	C28—C27—C26	119.6 (3)
C29—O7—C33	113.9 (3)	O6—C28—C27	124.3 (3)
C30—O8—C34	116.6 (3)	O6—C28—C29	115.3 (3)
N1—C1—C2	123.4 (3)	C27—C28—C29	120.4 (3)
C1—C2—C3	118.6 (3)	O7—C29—C28	120.9 (3)
C2—C3—C4	119.2 (3)	O7—C29—C30	119.5 (3)
C5—C4—C3	119.0 (3)	C28—C29—C30	119.5 (3)
N1—C5—C4	121.6 (3)	O8—C30—C31	125.0 (3)
N1—C5—C6	115.6 (3)	O8—C30—C29	114.8 (3)
C4—C5—C6	122.8 (3)	C31—C30—C29	120.2 (3)
N2—C6—C7	123.7 (3)	C26—C31—C30	119.4 (3)
N2—C6—C5	114.3 (3)	O10—C36—C37	102.0 (6)

### Hydrogen-bond geometry (Å, º)

**Table:**

*D*—H···*A*	*D*—H	H···*A*	*D*···*A*	*D*—H···*A*
C16—H16*C*···O5	0.96	2.58	3.097 (7)	114
C17—H17*B*···O8^i^	0.96	2.59	3.457 (6)	150
C18—H18···O3^ii^	0.93	2.42	3.100 (5)	130
C24—H24*B*···O7^iii^	0.96	2.55	3.414 (5)	149
C24—H24*C*···O1^iv^	0.96	2.38	3.281 (4)	157
C33—H33*B*···O6	0.96	2.54	3.075 (5)	115

Symmetry codes: (i) −*x*+1, −*y*+1, −*z*; (ii) −*x*, −*y*, −*z*+1; (iii) *x*−1, *y*, *z*; (iv) −*x*, −*y*+1, −*z*.
